# Spatial impacts of a multi-individual grave on microbial and microfaunal communities and soil biogeochemistry

**DOI:** 10.1371/journal.pone.0208845

**Published:** 2018-12-12

**Authors:** Sarah W. Keenan, Alexandra L. Emmons, Lois S. Taylor, Gary Phillips, Allison R. Mason, Amy Z. Mundorff, Ernest C. Bernard, Jon Davoren, Jennifer M. DeBruyn

**Affiliations:** 1 Department of Biosystems Engineering and Soil Science, University of Tennessee, Knoxville, Tennessee, United States of America; 2 Department of Anthropology, University of Tennessee, Knoxville, Tennessee, United States of America; 3 Department of Entomology and Plant Pathology, University of Tennessee, Knoxville, Tennessee, United States of America; 4 Bode Cellmark Forensics, Lorton, Virginia, United States of America; Oakland University, UNITED STATES

## Abstract

Decomposing vertebrates, including humans, result in pronounced changes in surrounding soil biogeochemistry, particularly nitrogen (N) and carbon (C) availability, and alter soil micro- and macrofauna. However, the impacts of subsurface human decomposition, where oxygen becomes limited and microbial biomass is generally lower, are far less understood. The goals of this study were to evaluate the impact of human decomposition in a multi-individual, shallow (~70 cm depth) grave on soil biogeochemistry and soil microbial and nematode communities. Three individuals were interred and allowed to decay for four years. Soils were collected from two depths (0‒5 and 30‒35 cm) along linear transects radiating from the grave as well as from within and below (85‒90 cm depth) the grave during excavation to assess how decomposition affects soil properties. Along radiating surface transects, several extracellular enzymes rates and nematode richness increased with increasing distance from the grave, and likely reflect physical site disruption due to grave excavation and infill. There was no evidence of carcass-sourced C and N lateral migration from the grave, at least at 30‒35 cm depth. Within the grave, soils exhibited significant N-enrichment (e.g., ammonium, dissolved organic N), elevated electrical conductivity, and elevated respiration rates with depth. Soil biogeochemistry within the grave, particularly in the middle (30‒35 cm) and base (70‒75 cm depth), was significantly altered by human decomposition. Mean microbial gene abundances changed with depth in the grave, demonstrating increased microbial presence in response to ongoing decomposition. Human-associated *Bacteroides* were only detected at the base of the grave where anoxic conditions prevailed. Nematode community abundance and richness were reduced at 70‒75 cm and not detectable below 85‒90 cm. Further, we identified certain *Plectus* spp. as potential indicators of enrichment due to decomposition. Here we demonstrate that human decomposition influences soil biogeochemistry, microbes, and microfauna up to four years after burial.

## Introduction

Decomposing vertebrates transform their surrounding environment by releasing compounds, particularly carbon (C) and nitrogen (N), [1–4] that stimulate invertebrate [5,6] and vertebrate scavengers [7], and alter surrounding physiochemistry and biological communities [2,8–11]. When carcasses decay in terrestrial ecosystems, the soil beneath exhibits a wide range of chemical changes, including increased concentrations of ammonium, dissolved organic carbon, dissolved organic nitrogen, phosphate, and calcium [4,10,12–15]. The lateral and vertical extent to which carcasses influence their surrounding environment depends on a variety of factors, including size of the animal and soil physical properties [16,17]. At the far end of the spectrum, for example, elevated soil nitrogen was detected up to 2 m away from decomposed muskox carcasses [18]. Prior studies of surface human decomposition have demonstrated that decomposition products migrate laterally through soil up to approximately 1 m away [12,19].

Changes in macro- and micro-fauna generally accompany the soil chemical changes in decomposition islands. Numerous insect and arthropod taxa change in response to carcass decomposition, with changes in community membership diagnostic of specific decay stages [20–22]. Soil microbial communities, both bacteria and fungi, also change during decay [9,10,23–25]: microbial activity peaks during active decomposition, and communities shift towards a higher proportion of anaerobic taxa such as Bacteroidetes and Firmicutes [10,24,26]. Such changes generally coincide with an overall decrease in diversity over time [27].

The majority of vertebrate decomposition studies in terrestrial ecosystems have focused on surface decomposition, over timescales ranging from days to years [12,18,28,29]. Surface decay rates are influenced by a range of variables that change on diurnal and seasonal scales, including temperature and precipitation or moisture [30–33]. In contrast, buried remains experience rapidly developing anaerobic conditions, limited gas and fluid exchange with surrounding soil, and greatly reduced insect and scavenger activity [34], resulting in a closed system relative to surface decomposition [14]. Additionally, the variable decomposition rates observed in burials [16,34] result in long periods before soft tissues are completely degraded. The contrast between surface and burial decomposition has also been noted in terms of impacts on microbial communities. For example, burial resulted in consistent measures of diversity with increases in richness and decreases in evenness compared to surface systems during decay [27].

The effects of buried carcass decomposition are more difficult to study, particularly with respect to identifying time-resolved changes, resulting in significant gaps in our understanding of subsurface decay processes in soil systems. Despite several prior studies of interred carcass decomposition [14,21,27,35,36], the potential for decomposing carcasses to influence soil physiochemistry and biology within and outside of a grave has not been fully explored. Additionally, the temporal duration that soils retain physiochemical indicators of decomposition is largely unknown. Several studies have examined changes to soil physiochemistry and microbial ecology after 1 to 1.2 years [35,36], and in both studies carcasses were still actively degrading. Assessing the impact of buried remains on soil chemistry and biology, and whether soils maintain chemical or biotic signatures of decay years post-deposition, after soft tissues have largely decomposed, may have applications in several areas: for example, insight into nutrient pulse or ‘hotspot’ dynamics in terrestrial ecosystems; a better understanding of taphonomy *i*.*e*. the interplay between environment and decomposing remains as they relate to long term preservation of remains; and/or determining the age of human graves and guiding recovery or discovery of clandestine graves.

Decomposition environments are host to multi-trophic level food webs, which are influenced by changes in soil physiochemistry [37–40]. Of the microfauna, free-living soil nematodes are of particular interest; nematodes are ubiquitous across all known ecosystems, comprising an estimated 80% of all multicellular organisms in the soil environment [41]. Differentiated feeding habits and successional patterns have rendered them useful as soil enrichment indicators [42–44]. Nematode enrichment has been observed in association with decaying carcasses [45], however limited work has been done to date in terms of a systematic description of nematode community responses to vertebrate decomposition [38,46]. At present, studies of microfaunal communities associated with decomposing vertebrate remains have been restricted to cataloging taxa of nematodes [38], testate amoeba [37,39], and arthropods [40,46] in surface soils. The effects of buried remains on subsurface microfaunal communities are poorly understood. Nematode community composition is sensitive to soil resource availability in agricultural systems [47–54] as well as physical or chemical disturbances [54–56]. Given the dynamic nature and high flux of nutrients to the soil during decay, it was expected that vertebrate decomposition would select for nematodes feeding on the bloom of decomposer bacteria and fungi associated with the carcass and/or tolerating the extreme physiochemical changes.

The goals of this study were to evaluate the spatial (vertical and lateral) changes in soil chemistry and biological communities, including microbes and nematodes, associated with a multi-individual grave, four years after burial of three human subjects. We expected to observe physiochemical indicators of decay in the soils within and immediately adjacent to the gravesite, including decreased pH, elevated electrical conductivity, elevated soil respiration rates, and elevated total C and N. In addition, we expected to observe enrichment of microbes and nematodes within the grave. Given the dense and clay-rich soil at the experimental site, lateral migration of porewater fluids transporting grave-derived organics was expected to be minimal and restricted to downward movement. The results provided novel insights into the impact of interred remains on soil biogeochemistry, and microbial and nematode ecology.

## Materials and methods

### Field location and multi-individual interment

The University of Tennessee Anthropological Research Facility (ARF) is the longest running human decomposition laboratory [2,57]. The ARF is located in Knoxville, Tennessee, United States on 2 acres of temperate mixed deciduous forest, with varying densities of understory and groundcover. The soil type consists of decomposed plant material and loam in the O-A horizons (0‒10 cm), underlain by clay loam and channery clay loam extending to bedrock (limestone, shale, and sandstone) [58]. In February 2013 two graves (~2 m x 2 m x 0.7 m) were excavated at the facility in an area that had not previously hosted decomposition experiments with an approximate slope of 1‒3% (S1 Fig). On 14 February 2013, three deceased human subjects (two white males and one white female, S1 Table) donated to the University of Tennessee Forensic Anthropology Center for the W. M. Bass Donated Skeletal Collection (http://web.utk.edu/~fac/collection.html) were placed into one of the graves. Individuals were stacked in the grave; the first individual placed (Individual A) was positioned in a W-E orientation, with the superior position in the W. The second individual, Individual B, was oriented N-S, and Individual C, the individual closest to the surface, was oriented W-E. The grave was backfilled and left undisturbed until March 2017. The second grave (referred to as the control grave) was excavated and backfilled at the same time as body placement and used as a negative control to simulate the effects of excavation in the absence of human decomposition. Because no living human subjects were involved in this research and no personally identifiable information was collected, the project was exempt from review by the University of Tennessee Institutional Review Board.

### Soil collection and chemical analyses

**Fig 1:**
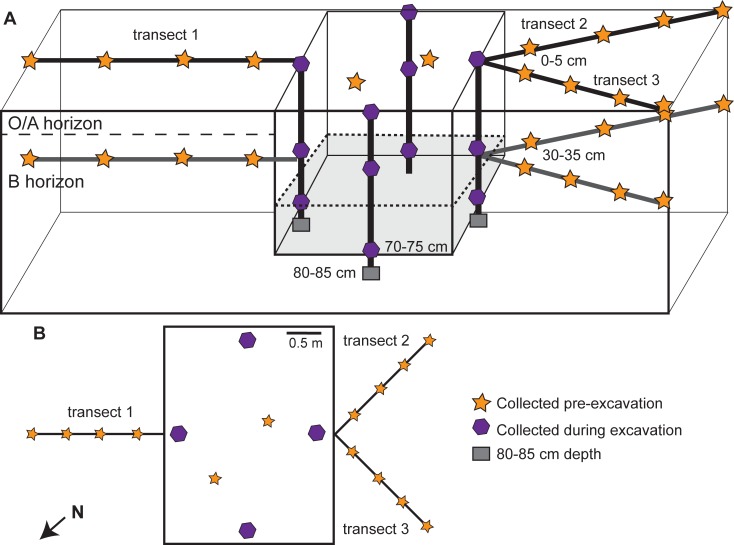
Soil sample locations within and outside of the multi-individual grave. (A) Cross-sectional view of sample locations. Samples were collected along three lateral transects (orange stars), within the disturbed grave soils (purple hexagons), and below the grave floor (gray squares) during excavation. Shading within the grave indicates the approximate depth of donor placement. Soils along lateral transects were collected from surface (0‒5 cm) and subsurface (30‒35 cm) soils. Soils within the grave were also collected at the base of the grave (70‒75 cm) and below the grave (85‒90 cm). (B) Top down map view of sampling locations and transect orientations.

The graves were excavated and human remains were removed in March 2017. At the time of disinterment, the individuals were in disparate states of decomposition. Skeletal elements from a single individual spanned multiple depths due to the nature of placement within the grave, and the grave was saturated with water, especially in the NW section. Individual C was skeletonized with essentially no adipocere or soft tissue present, while Individual B was partially skeletonized but with greater amounts of adipocere and some large portions of skin and soft tissue present, especially in the upper torso, hands, and feet. Conversely, individual A was nearly intact but in a suspended state of decomposition due to extensive adipocere formation, with pink muscle tissue and skin still present on most of the body. The torso (upper and lower) was encased in an outer shell of adipocere, which protected the underlying decomposing muscle and tissue. Soil samples were collected from three linear transects radiating from the grave, from inside the grave (Fig 1), and from below the grave once remains were recovered. The transect soils were collected on 14 February 2017, exactly 4 years after the bodies were interred and prior to grave excavation. The three linear transects (Fig 1) radiating 2 m from the grave were subsampled at 0.5 m intervals from the O/A horizon (0‒5 cm) and B horizon (30‒35 cm). Transects were selected based on logistical constraints (i.e., limestone boulders, fallen or live trees) and avoided any nearby gravesites. All transects were a minimum of 3 m from any nearby graves, and there are no records of previous surface vertebrate decomposition in these areas. Soil samples were collected using a 10 cm-diameter auger. Holes from O/A horizon surface samples were extended to 30 cm depth to allow collection of B horizon samples from 30‒35 cm with a 3 cm-diameter auger. Two O/A horizon, pre-excavation soil samples were collected from the top of the grave. Control soils were also collected from two regions prior to grave excavations. Three cores were obtained and composited from 0‒5 cm and 30‒35 cm from the control grave. Additional off-grave control cores (n = 3) were collected from the same depths at three undisturbed locations approximately 5 m from the grave.

During excavation (8‒9 March 2017), four additional O/A horizon samples were collected from the grave surface. Once excavations reached the human remains, three soil samples were collected immediately adjacent to the bodies at 30‒35 cm. An additional soil sample containing adipocere and other partially decomposed organic material was obtained from within the rib cage of Individual C (~40 cm depth). Following the complete removal of skeletal remains (14 March 2017), four soil samples were collected at the base of the grave (70‒75 cm) and three samples were collected from the 85 cm depth (15 cm below the base of the grave; the deepest sample possible due to the dense clay and proximity to the underlying bedrock). All samples (n = 45) were homogenized by hand in the field to remove rocks or vegetation greater than 2 mm prior to transport to the lab. Approximately 10 g of soil was flash-frozen in liquid nitrogen and stored at -80°C for DNA extraction. Soils for enzyme assays were weighed into sterile 15 mL tubes and frozen at -20°C until analysis.

All soil biogeochemical analyses were conducted within 24 hours after storing soils under field conditions overnight. Soil pH and electrical conductivity were measured using a 1:2 soil to deionized water slurry and an Orion Star A329 multiparameter meter (ThermoScientific). Soil gravimetric moisture was assessed in triplicate by oven-drying soil aliquots for at least 48 hours at 105°C. Respiration rates were measured using field-moist soils sealed in 60 mL serum bottles. Measurements immediately after capping (time 0) and after 24 hours of incubation at room temperature were conducted in duplicate using a LI-820 CO_2_ analyzer (LI-COR) with manual injection (0.5 mL volume). For soil extracts, field moist soils were incubated at room temperature on a shaking platform (150 rpm) in 0.5 M K_2_SO_4_ for four hours (soil:solution ratio of 1:4). Jars were allowed to sit for one hour to allow sediment to settle from solution. Soil slurries were then vacuum filtered (Ahlstrom, glass microfiber filters, 1 μm), and filtered extracts were stored at -20°C.

Ammonium concentrations in soil extracts were quantified following a microplate protocol after 2 hours of incubation, with minor modifications [59]. The ammonium standard [(NH_4_)_2_SO_4_] was dissolved in 0.5 M K_2_SO_4_ instead of deionized water to account for potential matrix effects. In addition, 70 μL (instead of 50 μL) of each soil extract or standard were pipetted into the microplate, and 50 μL of deionized water was used (instead of 100 μL) [59].

Soil nitrification potential was determined with a modified chlorate block method designed for a microplate reader [60–62]. The nitrification potential medium contained: 4 mL of 0.2 M K_2_HPO_4_, 0.5 mL of 0.2 M KH_2_PO_4_, 2.5 mL of 0.2 M (NH_4_)_2_SO_4_, and 10 mL of 1 M NaClO_3_, diluted up to 1 L with sterile deionized water. The color reagent consisted of 87.6 mL of sterile deionized water, 17 mL of 85% H_3_PO_4_, 4 g of sulfanilamide, and 0.1 g of N-(1-Naphthyl) ethylenediamine). A 100 ppm-N as NO_2_^-^ standard was made using NaNO_2_. Soils (2 g) were frozen at -20°C prior to beginning nitrification potential assays, which may underestimate the total amount of nitrite in the soil by 27% [61]. Nitrification potential medium (2 g soil: 14 mL medium) was added to each 50 mL tube after allowing the soil to fully thaw to room temperature [60]. Samples were vortexed (10 seconds) to break up soil aggregates. Tubes containing the chlorate-soil slurry were placed horizontally on a shaking incubator set to temperatures that corresponded to ambient conditions in the field and were allowed to shake for 5 minutes (120 rpm) before transferring 1 mL aliquot of slurry (T_0_) to sterile 1.5 mL microcentrifuge tubes. Tubes were placed back on the shaking platform, and subsampled again after 2.5 (T_1_) and 5 hours (T_2_). The tubes containing solution aliquots were immediately centrifuged for 2 minutes at 10,000 × g. Nitrite concentrations were determined (in triplicate) colorimetrically using 50 μL of the NO_2_^-^ color reagent and 200 μL of the supernatant from centrifuged samples in a 96-well plate. Absorbance values were measured with a plate reader at 543 nm after incubating for at least 10 minutes at room temperature.

Extracts were oxidized to quantify dissolved organic carbon (DOC) and dissolved organic nitrogen (DON) following the persulfate method [63]. DOC was measured as the amount of CO_2_ released after overnight incubation at 80°C. Nitrate concentrations of soil extracts and persulfate oxidized extracts (DON) were determined colorimetrically in triplicate for each sample after 5 hours of incubation at room temperature [64].

Soils previously frozen at -20°C were allowed to thaw to room temperature and were then used to assay extracellular enzyme activity. Potential activity of leucine aminopeptidase (LAP), N-acetyl-β-glucoseaminidase (NAG), and β-D-cellulobiosidase (CB) [65], as well as phosphodiesterase (PDE) [66] and collagenase (COL) were assayed using a 50 mM Tris buffer at pH 6.7, optimized based on average soil pH. The COL assay was developed from the EnzChek gelatinase/collagenase kit (Molecular Probes) and optimized for soils. The same soil slurry used to measure LAP, NAG, CB, and PDE was used to measure COL. A ratio of 20 μL soil slurry: 20 μL fluorescence substrate: 160 μL 1X reaction buffer was found to work best in this soil type for the COL assay. The fluorescence substrate, DQ gelatin, was diluted to a final working concentration of 80 μg mL^-1^. Standard curves were generated for each soil sample by spiking the soil with the kit-supplied *Clostridium histolyticum*, which has a known collagenase activity, with standard curves ranging from 0.01 to 1.0 U mL^-1^. Each standard contained 20 μL soil slurry: 20 μL standard: 20 μL fluorescence substrate: 140 μL 1× reaction buffer. Samples were loaded directly into an opaque 96 well plate, and fluorescence was measured on a Synergy H1plate reader (BioTek) after 3 hours.

### Soil biological community assessment methods

DNA was extracted from soils that had been stored at -80°C using the DNeasy Powerlyzer Powersoil kit (QIAGEN Inc.) per manufacturer’s instructions, following recommendations for clay soils. 250 mg of soil were used for each extraction. Soil DNA extracts were stored at -20°C. All soil DNA extracts were quantified for total DNA concentration using the Quant-iT PicoGreen dsDNA Assay Kit (Invitrogen) on a 96-well microplate reader using reduced assay volumes of 200 μL. Samples were quantified in duplicate while standards were run in triplicate. Five standards were included in each plate ranging from 0 μg mL^-1^ to 0.5 μg mL^-1^.

As a proxy for bacterial and fungal abundances, qPCR was used to quantify 16S rRNA and ITS gene abundances in soil using the Femto Bacterial DNA Quantification Kit and the Femto Fungal DNA Quantification Kit, respectively. Assays were performed in accordance with the manufacturer’s instructions using a CFX Connect Real-Time PCR Detection System (BioRad). Samples were quantified in triplicate, while standards were quantified in duplicate. A minimum of three no-template controls were included in each 96-well plate. Data are presented as copy number per gram of dry weight soil (copy gdw^-1^).

Human-associated *Bacteroides* are common gut microbes that have been found to enter the soil and persist during decomposition [10]. DNA extracts from all samples were used to test for the presence of human-associated *Bacteroides* (HuBac) via a qPCR assay developed by Layton et al. [67]. A dilution series of plasmid DNA containing the human-associated *Bacteroides* 16S rRNA gene [67] ranging from 10^7^ to 10^2^ copies per μL was used to generate a standard curve. Each 25 μl reaction contained 12.5 μl 2X TaqMan Universal PCR master mix (ThermoFisher), 6.5 μl nuclease-free water (ThermoFisher), 1.5 μl each of HuBac566f primer (10 μM) and HuBac692r primer (10 μM), 0.5 μl of HuBac594Bhqf probe (10 μM) (synthesized by Eurofins), and 2.5 μl of template DNA. The reactions were run on a CFX Connect (BioRad) using the following amplification conditions: 50°C for 2 minutes, 95°C for 10 minutes, and 40 cycles of 95°C for 30 seconds and 60°C for 45 seconds. Data are presented as copies per gram dry weight soil.

Nematodes were extracted from fresh soil using a sugar flotation-centrifugation method [68]. Total abundances (N) were expressed as nematodes per 100 cm^3^ soil. Nematodes were counted and identified to genus with the aid of a differential interference contrast (DIC) microscope. For uncertain identifications, Bongers [42], Geraert [69], Siddiqi [70], and various recently published keys and revisions were consulted. *Anaplectus* and *Plectus* spp. were identified according to Allen and Noffsinger [71], Andrassy [72], and Zell [73]. Nematodes were assigned trophic classifications based on identification: bacterial feeders (B), fungal feeders (F), plant parasites (Pp), predators (Pr), and omnivores (O) [74]. They were also assigned colonizer-persister (c-p) values, ranging from 1 through 5, representing a range between quickly reproducing enrichment opportunists associated with ephemeral nutrient sources or disturbed environments, to those that reproduce slowly and in low density, found most often in mature, stable environments [42]. Trophic groups and c-p classes were combined into functional guilds according to Bongers and Bongers [75]. Community richness was defined as the number of unique genera. Shannon diversity (H) and equitability (evenness, EH) were calculated using Eqs (1) and (2), respectively.

$$H=-\sum p_{i}\;(\ln p_{i})$$ (1)

EH=H/Hmax (2)

Where p_i_ is the proportion of the *i*^th^ genus in the sample community, and *Hmax* = *ln(n*), where *n* consists of the total number of genera in a sample [76]. Faunal indices were calculated according to [77], and consist of an enrichment index (EI) (3) and structure index (SI) (4), designed to characterize nematode communities based upon community structure and prevailing food web conditions.

EI=100(e/(e+b)) (3)

SI=100(s/(s+b)) (4)

Where *b* = ∑ *k*_*b*_*n*_b_, *k*_*b*_ is the weighting constant assigned to functional guilds B2 and F2, indicators of basal soil states, and *n*_*b*_ is the number of nematodes present in those guilds. Similarly, *e* is calculated with weighting constants assigned to functional guilds B1 and F2, indicators of enrichment, and *s* is calculated with weighting constants assigned to structure (B3‒5, F3‒5, O3‒5, Pr3‒5). EI (3) and SI (4) values are plotted on a faunal ordination diagram [77]. The Maturity Index (MI) (5) [42] consists of the weighted mean of all c-p values, excluding plant-parasitic taxa, and serves as a general indicator of soil enrichment status.

$$MI=\sum v_{i}*f_{i}$$ (5)

Where *v*_*i*_ is the c-p value (1‒5), and *f*_*i*_ is the frequency of each c-p class represented in the sample.

### Grave aqueous geochemistry

A perched water lens formed along the base of the grave. Cores from the 85-cm depth were collected from areas without standing water. A water sample was collected during excavation, stored overnight at 4°C, and filtered to 0.2 μm into Nalgene bottles. A subsample was filtered into an HCl-washed Nalgene bottle, and preserved by the addition of trace grade nitric acid for cation analyses. Anions and cations were analyzed using Ion Chromatography (Dionex ICS-2000 and 2100, respectively) at the University of Tennessee. Total alkalinity (as bicarbonate, HCO_3_^-^) was measured by titration with 0.1 N H_2_SO_4_ to pH 4.3 [78].

### Data analyses and statistics

To assess potential for lateral transport, soils collected along linear transects radiating from the grave at 0‒5 and 30‒35 cm depths were compared with soils collected from these same depths within the grave to evaluate potential changes in soil chemistry and biology. To determine the potential for vertical changes in soil chemistry within the grave, soils collected from 0‒5, 30‒35, 70‒75, and 85‒90 cm within the grave were compared. Soil chemical and biological data are presented on a gram dry weight (gdw) basis when appropriate. Significant differences between samples at depth and along transects were based on a one-way analysis of variance (ANOVA) followed by a Holm-Sidak post hoc test using SigmaPlot (version 14, Systat Software, Inc.). To evaluate the effects of depth and distance along the transect, two-way ANOVAs were also computed. For all ANOVAs, significance was set at p < 0.05.

Nematode community analyses were performed using the basic statistical functions in R (version 3.3.2), along with the “car” statistical package (version 2.1–4) [79,80]. For transect samples, in order to evaluate the effects of depth, transect distance, and interactions between factors, a two-way ANOVA was performed followed by a Tukey HSD posthoc test to identify differences.

To visualize the overall differences in soil biogeochemistry between all samples, a principal components analysis (PCA) was run on a Euclidean distance matrix of all samples (except for the rib cage sample, which was a strong outlier), including all physicochemical parameters measured along with biological parameters of activity (respiration, enzyme activities) and microbial gene abundances. The PCA was done in Primer 7 v 7.0.13 (PRIMER-e, Quest Research Ltd.).

## Results

### Lateral changes in soil biogeochemistry

In soils radiating laterally from the grave, gravimetric moisture was significantly elevated in surface samples (0‒5 cm) compared to their depth equivalent (30‒35 cm) along the transects (p < 0.001; F = 36.600), and gravimetric moisture significantly increased with distance from the grave (p < 0.001; F = 12.480). Surface soil pH (6.68 to 6.82) was not significantly different from soil pH at depth (6.05 to 6.74) (Table 1, S2 Table), and there were no significant differences in pH with proximity to the grave (Table 2). Conductivity was significantly elevated in surface soils compared to samples at depth (p < 0.001; F = 18.995). In surface soils, conductivity ranged from 18.26 to 30.75 μS cm^-1^, with lowest conductivity values observed in the top of the grave. At depth, values ranged from 10.91 to 20.75 μS cm^-1^, with highest conductivity values observed inside the grave (Table 1, S2 Table).

**Table 1:** Measured soil physicochemical parameters, presented as means and standard deviations of transects (distances of 0.5, 1, 1.5, 2 m) and grave soils (distance of 0 m). n = 3 samples from three independent transects or 3 grave soil samples. DON = dissolved organic nitrogen; DOC = dissolved organic carbon. * = sample collected from the ribcage.

Distance from grave (m)	Depth (cm)	Gravimetric moisture	pH	Conductivity (μS cm^-1^)	Ammonium (μg NH_4_-N gdw^-1^)	Nitrification potential rate (mg NO_2_-^-^N gdw^-1^ day^-1^)	CO_2_ released (μg C gdw^-1^ day^-1^)	DOC (mg CO_2_ gdw^-1^)	Nitrate (μg NO_3_-^-^N gdw^-1^)	DON (μg N gdw^-1^)	Bacterial gene copies (x 10^8^) gdw^-1^	Fungal gene copies (x 10^8^) gdw^-1^
2	0–5	0.377 ± 0.03	6.75 ± 0.25	29.92 ± 9.75	7.39 ± 1.8	0.126 ± 0.10	30.9 ± 5.6	2.94 ± 0.6	3.31 ± 2.5	11.31 ± 0.8	125 ± 19	5.3 ± 3.8
2	30–35	0.309 ± 0.02	6.05 ± 0.77	10.91 ± 2.83	2.54 ± 1.2	0.039 ± 0.03	6.18 ± 1.2	5.26 ± 6.2	0.451 ± 0.21	12.69 ± 8.3	28.5 ± 39	3.5 ± 3.0
1.5	0–5	0.397 ± 0.04	6.82 ± 0.19	28.37 ± 9.83	9.00 ± 4.7	0.174 ± 0.14	37.6 ± 6.9	3.09 ± 0.9	3.34 ± 1.3	16.41 ± 6.3	134 ± 76	5.5 ± 3.9
1.5	30–35	0.335 ± 0.03	6.28 ± 0.73	13.3 ± 7.15	2.86 ± 0.6	0.038 ± 0.03	10.1 ± 0.8	3.52 ± 2.0	0.606 ± 0.35	8.14 ± 2.1	57.2 ± 43	4.1 ± 4.9
1	0–5	0.374 ± 0.02	6.68 ± 0.22	30.75 ± 12.05	5.75 ± 0.5	0.120 ± 0.05	31.0 ± 8.9	2.16 ± 0.5	3.74 ± 2.3	10.19 ± 1.2	123 ± 64	5.8 ± 2.7
1	30–35	0.267 ± 0.03	6.60 ± 0.67	20.75 ± 1.36	2.75 ± 0.3	0.023 ± 0.01	6.78 ± 2.5	2.42 ± 1.8	0.708 ± 0.23	5.09 ± 0.3	37.1 ± 14	2.2 ± 0.56
0.5	0–5	0.315 ± 0.02	6.77 ± 0.22	27.04 ± 9.61	5.57 ± 2.0	0.053 ± 0.03	22.6 ± 8.7	1.54 ± 0.5	2.65 ± 1.1	10.03 ± 3.6	127 ± 32	8.3 ± 1.3
0.5	30–35	0.236 ± 0.01	6.74 ± 0.48	12.74 ± 1.75	2.99 ± 1.3	0.036 ± 0.03	8.39 ± 3.2	0.99 ± 0.5	0.943 ± 0.48	5.02 ± 1.0	25.6 ± 17	2.3 ± 1.8
0	0–5	0.321 ± 0.01	6.40 ± 0.38	18.26 ± 7.35	4.23 ± 1.8	0.011 ± 0.04	20.0 ± 7.7	2.18 ± 0.6	2.23 ± 0.6	7.51 ± 1.4	40.2 ± 19	5.9 ± 2.1
0	30–35	0.375 ± 0.02	6.21 ± 0.13	19.48 ± 10.7	3.84 ± 1.4	0.040 ± 0.01	21.0 ± 21	1.93 ± 0.8	3.55 ± 2.1	9.98 ± 2.3	21.0 ± 26	21 ± 35
0	40*	0.974	6.36	48.57	14.181	0.227	273	5.50 ± 0.6	0.839	22.62	629 ± 12	796 ± 18
0	70–75	0.464 ± 0.04	7.20 ± 0.51	134.8 ± 38.1	742 ± 389	-0.061 ± 0.03	83.5 ± 29	6.96 ± 4.6	1.04 ± 1.1	126.1 ± 37	26.2 ± 17	20 ± 13
0	85–90	0.335 ± 0.02	7.66 ± 0.36	139.9 ± 16.4	1075 ± 390	0.006 ± 0.03	23.6 ± 2.9	5.19 ± 2.5	3.22 ± 3.0	110.4 ± 13	0.51 ± 0.7	0.02 ± 0.03
Control (off-grave)	0–5	0.476	7.04	40.40	8.043	0.569	38.4	4.43	5.530	16.490	234 ± 2.4	79 ± 0.42
Control (off-grave)	30–35	0.312	7.47	21.06	2.636	0.048	7.72	1.09	0.851	6.174	29 ± 0.32	1.9 ± 0.061
Control grave	0–5	0.342	6.66	14.19	4.956	0.040	31.7	1.60	0.949	7.632	101 ± 1.2	6.0 ± 0.23
Control grave	30–35	0.309	6.30	4.316	2.099	0.054	5.56	2.13	0.947	7.016	16 ± 0.096	1.7 ± 0.009

**Table 2:** The effects of depth and distance on soil physiochemistry and biology. Results of ANOVAs comparing depths or five distances (0, 0.5, 1.0, 1.5, and 2.0 m), as well as interaction effects. Significant differences as a function of depth or distance from the grave are bolded (based on one-way ANOVAs; p < 0.05). Rates are presented per gram dry weight (gdw). PDE = phosphodiesterase activity; NAG = N-acetyl-β-glucoseaminidase activity; CB = β-D-cellulobiosidase activity; LAP = leucine aminopeptidase activity; COL = collagenase activity.

	Depth		Distance		Depth*Distance	
	p	F	p	F	P	F
Gravimetric moisture	**<0.001**	36.6	**<0.001**	12.48	**<0.001**	16.45
pH	0.07	3.61	0.39	1.08	**<0.001**	14.47
Conductivity (μS cm^-1^)	**<0.001**	19.00	0.535	0.80	**<0.001**	38.57
Ammonium (μg NH_4_-N gdw^-1^)	**<0.001**	24.33	0.417	1.02	**<0.001**	16.06
Nitrification potential rate (mg NO_2_-^-^N gdw^-1^ day^-1^)	**0.008**	8.47	0.14	1.93	**0.010**	7.34
Respiration (μg C gdw^-1^ day^-1^)	**<0.001**	32.20	0.588	0.72	0.081	3.24
Dissolved organic carbon (DOC) (mg CO_2_ gdw^-1^)	0.557	0.36	0.177	1.73	0.620	0.25
Nitrate (μg NO_3_-^-^N gdw^-1^)	**<0.001**	14.55	0.528	0.82	**0.002**	12.05
Dissolved organic nitrogen (DON) (μg N gdw^-1^)	**0.009**	8.13	**<0.001**	15.29	**<0.001**	22.13
PDE (nmols hr^-1^ gdw^-1^)	0.243	1.438	0.838	0.354	0.211	1.62
NAG (nmols hr ^-1^gdw^-1^)	**0.014**	7.089	0.078	2.417	**0.003**	9.80
CB (nmols hr^-1^ gdw^-1^)	**<0.001**	24.336	**0.031**	3.225	**<0.001**	23.52
LAP (μmols hr^-1^ gdw^-1^)	**<0.001**	61.319	**0.016**	3.833	**<0.001**	44.95
COL (U hr^-1^ gdw^-1^)	0.386	0.779	0.136	1.953	0.648	7.22
Bacterial copy numbers gdw^-1^	**<0.001**	31.278	**0.039**	3.005	**<0.001**	15.15
Fungal copy numbers gdw^-1^	0.901	0.0159	0.393	1.071	0.520	0.422
Total DNA (ng gdw^-1^)	**<0.001**	30.334	0.053	2.738	**<0.001**	18.93

**Fig 2:**
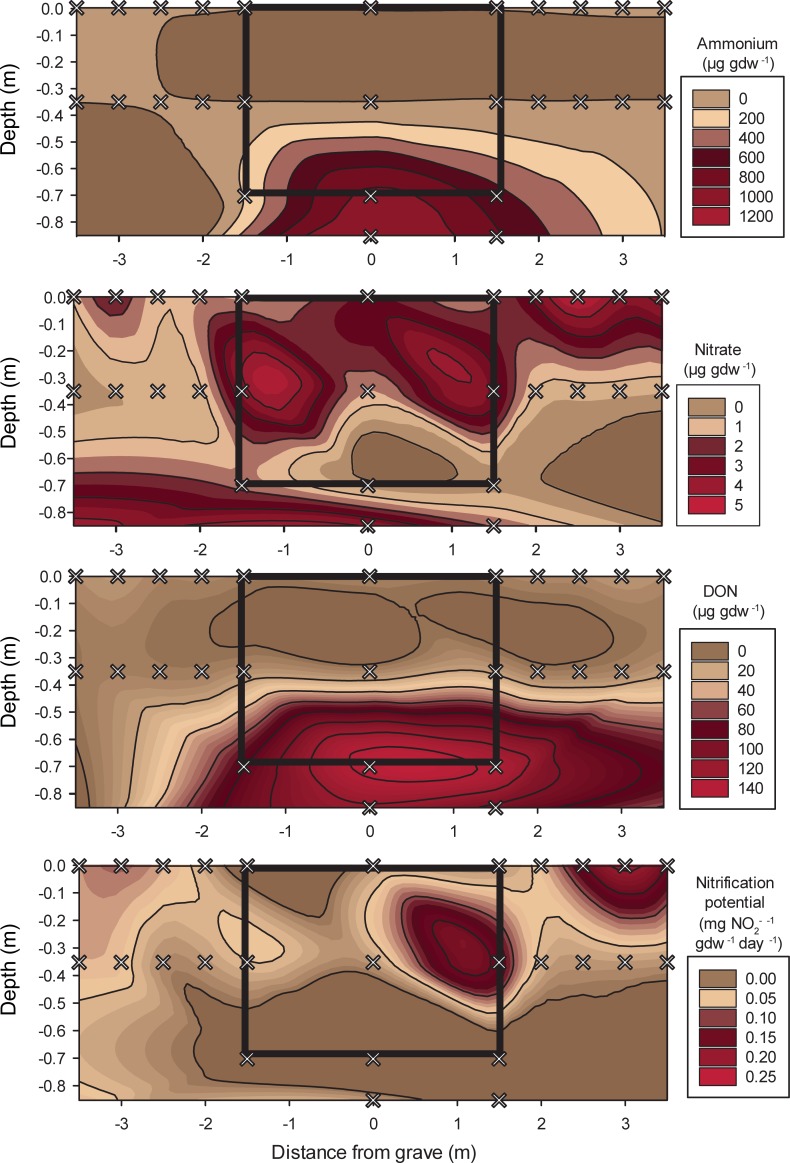
Cross-sectional contour maps of nitrogen concentrations or flux rates measured along lateral transects and within the multi‒individual grave. Grey x indicate soil sampling locations. The black box in each figure outlines the excavated area of the grave.

In general, nitrogen pools were elevated in surface soils compared to their depth equivalents along transects (Fig 2). Mean ammonium concentrations were 6.03 ± 2.7 μg NH_4_-N gdw^-1^ in surface soils and decreased to 2.80 ± 1.2 μg NH_4_-N gdw^-1^ at depth (p < 0.001; F = 24.326). Interestingly, we did not observe a significant enrichment of NH_4_^+^ closer to the grave. The highest ammonium concentrations were measured in surface soils at 1.5 m and 2.0 m from the grave, but these differences were non-significant. Nitrate decreased from a mean of 2.92 ± 1.5 to 1.17 ± 1.4 μg NO_3_-^-^N gdw^-1^ (p < 0.001; F = 14.6) between surface samples and those collected at depth. Nitrate was significantly elevated in surface soils compared to their depth equivalents at 1.0, 1.5, and 2.0 m along transects. At 0 and 0.5 m, [NO_3_^-^] was similar in surface and subsurface samples. Soils collected from the grave had greater [NO_3_^-^] at 30‒35 cm depth compared to grave surface soils. In contrast, the opposite trend was observed in transect soils, where [NO_3_^-^] was greater in surface soils and comparatively depleted at 30‒35 cm depth (Tables 1 and 2). Dissolved organic nitrogen (DON) increased with distance from the grave (p < 0.001; F = 15.290) and was elevated in surface compared to subsurface soils (p = 0.009; F = 8.130) along the transects. Similar to NO_3_^-^, DON was elevated at the 30‒35 cm depth and relatively depleted in the grave surface soils. Nitrification potential rates decreased with depth (p = 0.008; F = 9.194), except for soils collected from the grave, which exhibited greater nitrification potential rates in the 30‒35 cm soils compared to the grave surface soil (Tables 1 and 2).

### Lateral changes in soil microbial and microfaunal communities

Microbial respiration rates, measured as CO_2_ produced during a 24 hour incubation at ambient temperature, were three times lower in deep samples compared to surface soils along the transects (p < 0.001; F = 32.2). Within the grave, respiration rates were highly variable at 30‒35 cm depth, ranging from 7.2 to 45.1 μg CO_2_-C released gdw^-1^ day^-1^. At 0 and 0.5 m along the transects, there was no significant difference between respiration rates in surface and 30‒35 cm soils. Respiration rates were significantly higher further from the grave (1.0, 1.5, and 2.0 m), however DOC did not significantly differ in surface and subsurface soil along the transects or as a function of distance from the grave (Tables 1 and 2).

**Fig 3:**
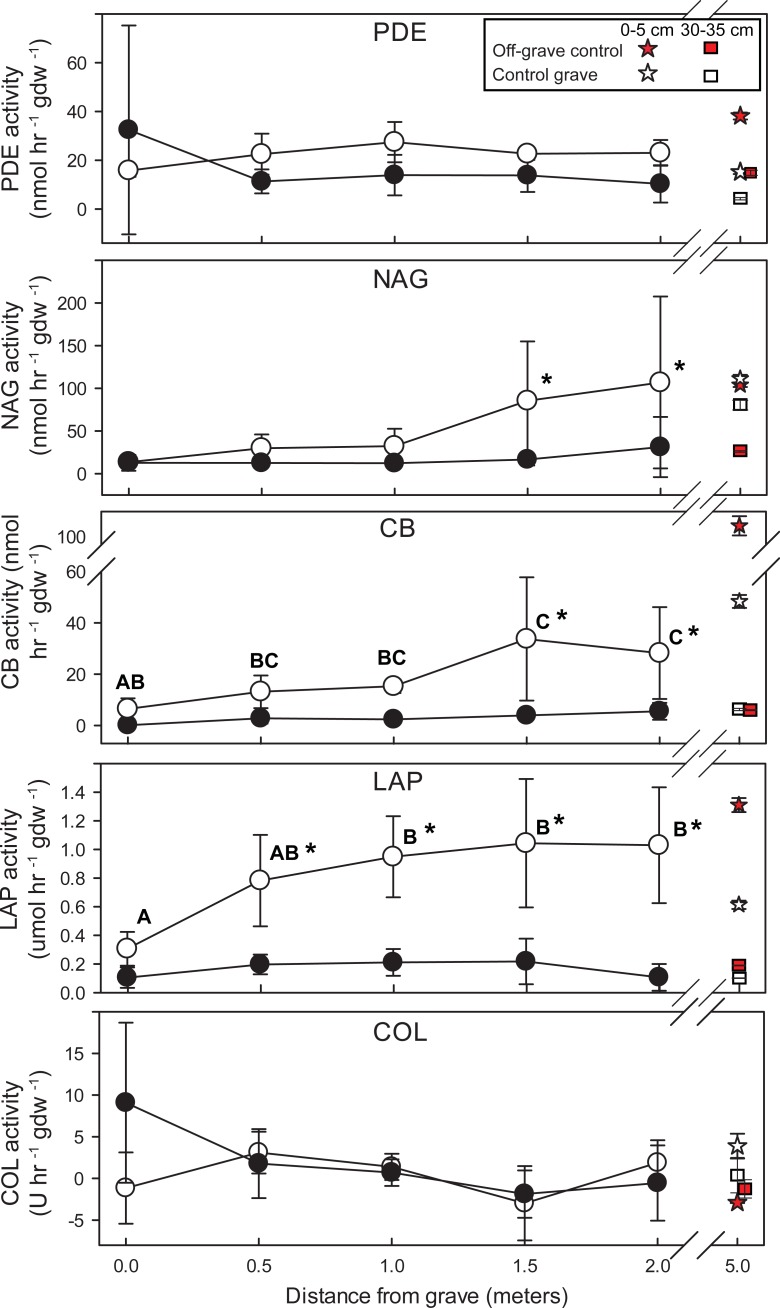
Potential extracellular enzyme rates along linear transects from the grave. Points are means of n = 3 samples from three independent transects. Significant changes in enzyme activity between distances are denoted by letters. Significant differences between surface soils (0‒5 cm, open circles) and soils collected at 30‒35 cm depth (filled circles) are indicated by asterisks. Measurements from off-grave site and control grave are included for reference. PDE = phosphodiesterase activity; NAG = N-acetyl-β-glucoseaminidase activity; CB = β-D-cellulobiosidase activity; LAP = leucine aminopeptidase activity; COL = collagenase activity.

The potential rates of several extracellular enzymes changed along the transects. Leucine amino peptidase (LAP), N‒acetylglucoseaminidase (NAG), and cellulobiosidase (CB) activities were higher in surface compared to depth (Table 2, S3 Table). CB and LAP activity increased with increasing distance from the grave (Fig 3). Phosphodiesterase (PDE) and collagenase (COL) did not differ significantly between depths or with distance from the grave (Table 2).

As a proxy for bacterial and fungal abundances, 16S rRNA and ITS gene copy numbers were quantified using qPCR assays. Bacterial gene copy numbers were elevated in surface soils compared to deep soils, and gene copy numbers increased in surface soils with distance from the grave relative to copy numbers observed within grave surface soils (p = 0.039, Tables 1 and 2). Within the grave, bacterial copy numbers significantly varied (p < 0.001) with a significant decrease in the 85‒90 cm layer (Table 1). Fungal copy numbers did not change significantly as a function of depth or distance along transects (Table 1). Using the qPCR assay specifically targeting human-associated *Bacteroides*, no human-associated *Bacteroides* were detected in any of the soils along the transects.

Along the transects, nematode abundances, richness and diversity were higher in surface soils compared to those at depth (Table 3). In surface soils along the transects, diversity, evenness, and MI did not significantly change with distance from the grave; however, richness increased with distance from the grave: samples from 1, 1.5, and 2.0 m had significantly higher richness than those collected from the surface of the grave. Evenness increased with distance from the grave in the subsurface (30 cm soils). Subsurface soils also exhibited significantly higher richness at distances of 1 and 1.5 m from the surface of the grave.

**Table 3:** Mean nematode abundance and diversity indices by depth (± standard deviation).

Location	Depth (cm)	Abundance	Richness (Genera)	Evenness	Shannon diversity	Maturity index (MI)
Transects (0.5–2 m distance)	0‒5*	**445.5 ± 191.0**^**A**^	**14.4 ± 3.6** ^**A**^	0.7 ± 0.1	1.9 ± 0.3	**1.9 ± 0.2** ^A^
	30‒35*	**148.3 ± 209.7**^**B**^	**8.9 ± 2.9** ^**B**^	0.8 ± 0.2	1.6 ± 0.6	**2.3 ± 0.4** ^B^
Grave (0 m distance)	0‒5	**480.6 ± 191.9** ^**A**^	**10.3 ± 2.0** ^**A**^	0.7 ± 0.1	1.7 ± 0.2	1.8 ± 0.2
	30‒35	**480.0 ± 306.2** ^**A**^	**7.7 ± 2.1** ^**AB**^	0.4 ± 0.2	0.8 ± 0.4	2.1 ± 0.1
	40‒45	**352.0**	**8.0**	0.8	1.7	1.5
	70‒75	**25.0 ± 26.1** ^**B**^	**4.0 ± 4.1** ^**B**^	0.7 ± 0.5	0.9 ± 0.9	1.7 ± 0.5
	85‒90	**0.0**	**0.0**	0.0	0.0	n/a

Significant differences between depths are indicated in bold and superscript letters designate groups that are not significantly different from each other (within either transects or grave) based on the Tukey HSD post hoc test.

*Data for transects represent the mean of samples from all distances (0.5, 1.0, 1.5 and 2.0 m) in all three transects (n = 18: 0‒5 cm, n = 15: 30‒35 cm), as there was no statistically significant difference with distance from grave.

**Fig 4:**
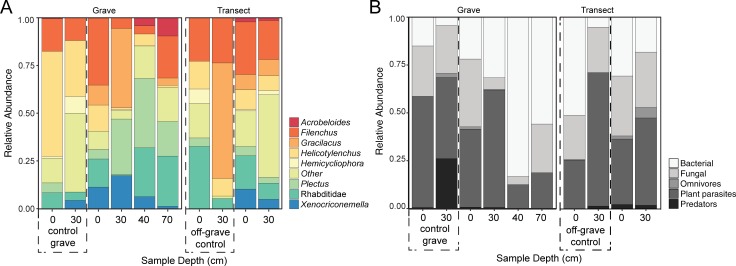
Nematode relative abundance and trophic groups by depth. (A) Relative abundances are based on taxa found in at least 50% of all soil samples. (B) Relative abundances of trophic groups are presented for all nematode taxa. Control grave (excavated, but no donors) and off‒grave controls (undisturbed site) are included for comparison. No taxa were recovered from the 85‒90 cm depth soils below the grave.

All surface transect soil samples contained the F2 fungal feeder *Filenchus* (mean of 28% relative abundance) and the B2 bacterial feeder *Plectus* (5%), with additional representation from bacterial feeding c-p classes 1‒3 (Rhabditidae, 18%; *Acrobeloides*, 2%; *Prismatolaimus*, 1%) and plant parasites (*Xenocriconemella*, 10%; *Gracilacus*, 8%; *Helicotylenchus*, 10%; *Meloidogyne*, 2%; *Xiphinema*, 2%) (Fig 4A). At the 30‒35 cm depth, *Filenchus* was reduced to 20%, but was present in all samples. *Meloidogyne* (11%), Rhabditidae (8%), *Gracilacus* (8%), *Helicotylenchus* (8%) and *Plectus* (3%) were also present at depth (Fig 4A). Between surface samples and those at depth, there was an overall shift from bacterial feeding (B) to plant-parasitic (Pp) taxa (Fig 4B, S4 and S5 Tables).

### Changes in soil biogeochemistry within the grave

The base of the grave contained a perched water lens that accumulated within and surrounded the bodies. As a result, soil gravimetric moisture differed significantly with depth (p < 0.001; F = 33.511); soil moisture increased with depth from the surface (0.321 ± 0.01) to the base of the grave (0.464 ± 0.04) where soils were saturated. Below the grave, soils were unsaturated with gravimetric soil moisture of 0.335 ± 0.02. Compared to the transects and control grave and off-grave controls, gravimetric moisture at 30‒35 cm was higher within the grave (Table 1). Soil pH was elevated at depth compared to surface samples, reaching a maximum of 7.66 ± 0.36 at 85‒90 cm depth. A similar pattern was observed with electrical conductivity, which was greatest in soils collected from 85‒90 cm (139.9 ± 16.4 μS cm^-1^) (Table 1, S2 Fig).

Ammonium concentrations increased several orders of magnitude with depth, reaching maximum concentrations at the 85‒90 cm depth (1075 ± 390 μg NH_4_-N gdw^-1^) (p < 0.001; F = 17.105) (Fig 2, Table 2). A similar pattern was observed with DON and DOC, both of which were elevated in the 70‒75 cm and 85‒90 cm samples (Fig 2, Table 1). Within the grave, nitrate concentrations were elevated but not significantly different at the 30‒35 cm depth (Fig 2). Nitrification potential rates decreased with depth (p = 0.009, F = 6.192) (Fig 2).

A single sample of partially decomposed organic material mixed with mineral soil was recovered from within the ribcage of Individual C at ~40 cm depth. The material had elevated conductivity and gravimetric moisture compared to soils collected adjacent to the body at 30‒35 cm depth (Table 1). Ammonium, nitrification potential rates, DOC, and DON were elevated in this sample as well. Respiration rates were an order of magnitude greater compared to soils collected anywhere else in the grave or along the linear transects.

The water sample collected from the perched groundwater at the base of the grave was pH 6.23 (at 11.5°C) with an electrical conductivity of 778.5 μS cm^-1^. The water contained: 17.48 mg L^-1^ Na^+^, 3.78 mg L^-1^ K^+^, 38.91 mg L^-1^ NH_4_^+^, 85.57 mg L^-1^ Ca^2+^, 6.43 mg L^-1^ Mg^2+^, 7.87 mg L^-1^ Cl^-1^, 11.67 SO_4_^2-^, and an alkalinity of 302.8 mg L^-1^ (as HCO_3_^-^). The grave water chemistry was similar to surface water in East Tennessee (S3 Fig), but contained significantly more ammonium than typical surface water, which ranged from 0.01 to 0.13 mg L^-1^.

### Changes in biological communities within the grave

**Fig 5:**
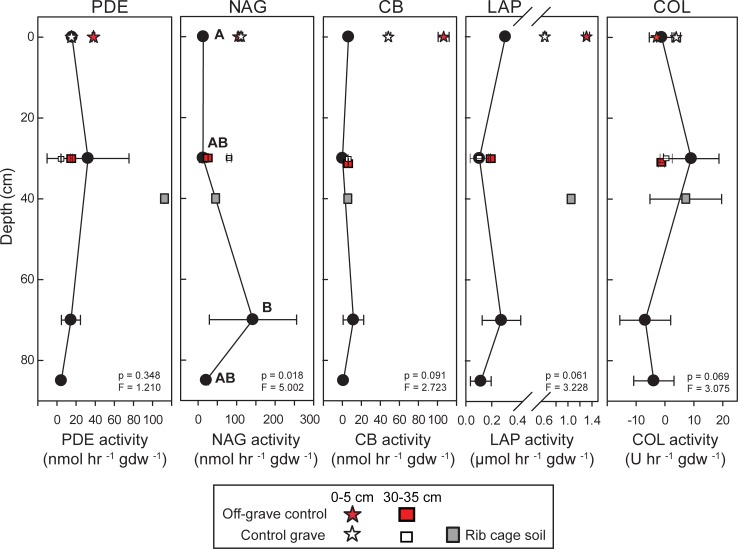
Extracellular enzyme activities within the multi-individual grave. Control grave and off-grave samples are included for reference. Significant differences in enzyme activity with depth are denoted by letters. PDE = phosphodiesterase activity; NAG = N-acetyl-β-glucoseaminidase activity; CB = β-D-cellulobiosidase activity; LAP = leucine aminopeptidase activity; COL = collagenase activity.

Microbial enzymatic activity did not significantly change with depth in the grave, except for NAG, which was an order of magnitude greater in the 70‒75 cm samples (p = 0.01, F = 5.002) (Fig 5).

Bacterial and fungal rRNA gene copy abundances were not significantly different through the grave; below the grave (85‒90 cm), abundances were significantly reduced. Human-associated *Bacteroides* were only detected in four of the 45 soil samples tested. These four samples were collected at the base of the grave (70‒75 cm), where *Bacteroides* gene abundances ranged from 4.9 x 10^4^ ± 1.9 x 10^4^ to 2.9 x 10^6^ ± 1.7 x 10^6^ copies gdw^-1^ (S4 Fig). It should be noted that this was not simply due to the amount of DNA extracted from soil (which could affect detection limits), as DNA extracted decreased significantly with depth, with less than 1 ng gdw^-1^ recovered at 85‒90 cm depth (S4 Fig).

Nematode abundances within the grave were similar between surface and 30‒35 cm depth soils, but were reduced in abundance at 70‒75 cm and not detectable below the base of the grave (85‒90 cm) (Table 3). Nematode community richness was significantly reduced at 70‒75 cm (Table 3). No significant differences in diversity, evenness or MI were detected within the grave (Table 3). Nematode community composition at the surface of the grave was similar to that found along transects (Fig 4). Taxa present in surface grave soils included: F2 fungal feeder *Filenchus* (35% of total abundance), B1-2 bacterial feeders Rhabditidae (15%) and *Plectus* (5%), and plant parasites *Helicotylenchus* (14%) and *Xenocriconemella* (11%) (Fig 4A). The plant parasite *Gracilacus* (10%) was present in most samples. A marked shift in community composition was observed at 30‒35 cm, where fungal-feeding taxa were present in lower proportions (*Filenchus*, 6%), and plant parasites were more abundant (*Gracilicus*, 42%; *Xenocriconemella*, 18%). Within bacterial feeding groups, B1 taxa predominated (Rhabditidae, 18%) in surface samples, and B2 taxa were more abundant at 30‒35 cm (*Plectus* 29%). One *Plectus* species identified was only found in grave samples. At 70‒75 cm depth, community membership decreased to only a few genera (Table 3, S6 Table) largely composed of B1‒2 (Rhabditidae, *Acrobeloides*, and *Plectus*), and F2 (*Filenchus*) taxa. The plectid component consisted of five species, according to Allen and Noffsinger [71], Andrassy [72], and Zell [73]. *Anaplectus granulosus* (Bastian), *Plectus cirratus* Bastian and *P*. *parietinu*s Bastian were commonly collected from most surface samples but were scarce or absent from deeper cores. *Plectus longicaudatus* Bütschli was frequently collected from the 30‒35 and 70‒cm samples, and was particularly abundant (80% of total nematodes) in soil associated with the 30-cm-deep water lens sample and in the ribcage sample. *Plectus aquatilis* Andrassy was collected only from the water lens sample. Tardigrades (*Hypsibius* sp.) were abundant in the water lens sample but infrequent in most other samples.

**Fig 6:**
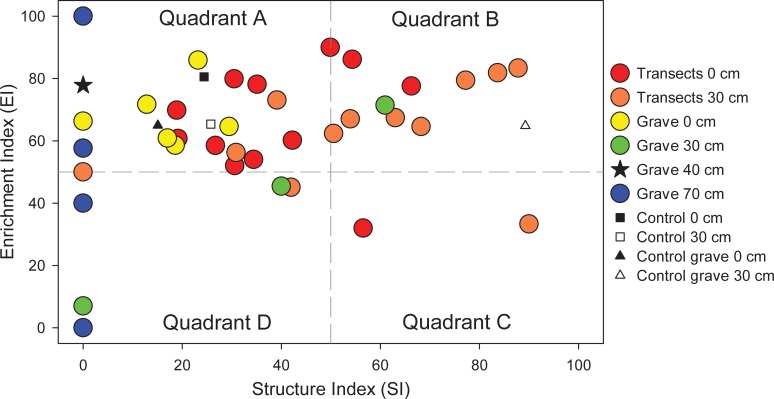
Faunal profiles representing the structure (SI) and enrichment (EI) conditions of the soil food web within the grave, along transects, and in control soils.

Nematode communities were examined using a faunal profile analysis, which shows nematode functional group responses (Fig 6). Communities in quadrant A are indicative of a response to a highly enriched and/or disturbed environment, with an increased relative abundance of B1 bacterial feeders. Communities within quadrant B also indicate enrichment, but with a more balanced ratio of bacterial and fungal feeders, as well as increased predatory and omnivorous taxa characteristic of a more stable environment. Quadrant C represents mature communities with a greater abundance of fungal feeders, often found in soils with low disturbance and high carbon:nitrogen ratios. Quadrant D is indicative of a community dominated by fungal feeders, often found in soils with depleted resources [77]. The faunal profile of this study shows that within 0‒5 and 30‒35 cm depths, both grave and transect communities largely occupy quadrants A and B, exhibiting similar degrees of enrichment but variable community structures, as shown on the structure index (SI) axis (Fig 6).

The organic matter-rich sample collected within the ribcage had elevated respiration, PDE and LAP activity compared to any other sample within the grave, and indicates elevated microbial activity. While LAP activity was comparable to activities measured in surface control grave and off-grave samples, PDE activity was higher than controls and bacterial and fungal gene copy numbers were higher than any other soil samples. Surprisingly, despite the fact that this sample was recovered from within the body cavity, human-associated *Bacteroides* were not detected. Nematode communities in this sample were not comparable to surrounding soil samples in all metrics (Table 3). The community was dominated by B1 and B2 bacterial feeders, primarily *Plectus* (36%), Rhabditidae (26%), and Diplogasteridae (15%); the last of which was not observed in any of the other samples in this study (Fig 4, S6 Table).

**Fig 7:**
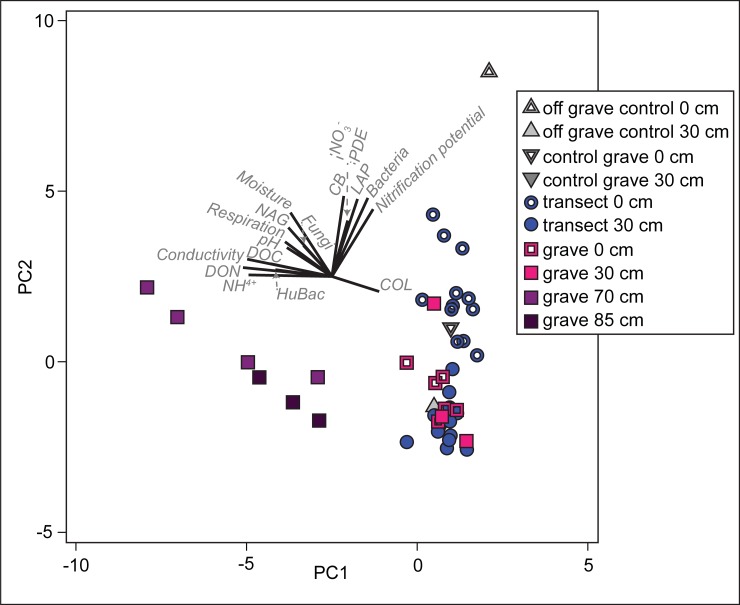
Principal components analysis of grave soils showing a clear difference in soil chemistry in samples from the bottom and below the grave. PC1 accounted for 33% of the variation; PC2 accounted for an additional 28.4%, for a cumulative variation explained of 61.4%.

When taken all together, the combination of soil physical, chemical, and biological data shows some overall patterns in these grave soils (Fig 7). The most impacted soils in this system were the within-grave samples at 30‒35, 70‒75 cm, and 85‒90 cm. While differences were expected between surface and deep samples along transects, these differences were not dissimilar from control sites and therefore we did not detect an overall impact of human decomposition on soils radiating laterally from the grave.

## Discussion

One of the goals of this study was to evaluate the potential for chemical inputs released by decomposing human subjects within a grave to influence soil biogeochemistry and biology within and outside of the grave four years post-burial. There are two processes that may explain the observed changes in soil properties in and around graves: (1) physical mixing and compaction of the soil during grave infill, and (2) human decomposition. In surface soils along lateral transects as well as within the grave, some of the observed physiochemical and biological changes may have been due to physical mixing and compaction. In particular, LAP and CB activities were lower in control grave samples compared to off-grave controls, suggesting that physical disruption of the soil has the potential to influence microbial enzymatic activity. Additionally, conductivity, ammonium, and nitrification potential rates were all lower in surface soils on the control grave compared to the off-grave control soils. This suggests that physical processes explain some of the observed differences, and four years after grave excavation and infill, surface soils have not returned to native conditions.

Despite some influence from physical processes, the overwhelming majority of changes observed within the grave at 30‒35 cm, 70‒75 cm, and 85‒90 cm depth were due to human decomposition. At 30‒35 cm depth, elevated nitrate and nitrification potential within the grave contrasted with soil collected outside of the grave (Fig 2), and indicates that soils contain enough oxygen to support nitrification. Fungal abundances were also elevated at the 30‒35 cm depth indicative of a microbial ‘bloom’. The presence of water-saturated soils from ~60 to 75 cm depth at the base of the grave resulted in suboxic to anoxic conditions; therefore it was not surprising to observe inhibited nitrification and a reduction in nematode abundances at these depths. At the base of the grave, ammonium, DON, DOC, and respiration rates were elevated, reflecting C and N compounds sourced from the bodies. Carcass-sourced organics were still present four years after burial, including soft tissue and adipocere formation on two of the donors. Adipocere is often observed in wet and anoxic environments with low gas diffusivity and low oxidation‒reduction potentials [16,81,82] and was therefore not a surprise in this environment. Moreover, reduced rates of LAP and PDE activity at depth, indicators of protein and nucleic acid degradation, suggest that oxygen limitation may be controlling microbial enzymatic activity despite the presence of labile C and N resources (i.e., remnant soft tissues). Elevated NAG activity at the base of the grave, an enzyme specific to chitin or fungal degradation, suggests that microbes are able to utilize a more recalcitrant C and N pool, and this activity is likely driven by anaerobic microbial communities. It is also notable that the saturated base of the grave was the only location where human-associated *Bacteroides* were detected, suggesting that the anoxic conditions and/or the presence of remaining soft tissue on the lowest body were conducive to long-term preservation of these obligate anaerobes. Our previous work showed that human-associated *Bacteroides* persisted for over a year in surface soils below decomposing donors, preserved by soft tissue remains that restricted oxygen flow to the soils [10]. Here we have added to this observation by showing that in saturated subsurface conditions, human-associated microflora can persist at least four years post-mortem.

We examined nematode communities associated with this multi‒individual grave in order to determine whether there was an influence of human decomposition on soil microfauna. Nematode communities are often used as indicators of disturbance or enrichment because of their successional patterns. For example, manure addition to agricultural fields causes initial blooms of enrichment opportunists followed by taxa less affected by chemical disturbance [83]. In this study, a minor increase in nematode richness was observed > 0.5 m away from the grave in surface soils. Grave surface soil richness was consistent with that found in grave controls. Since the grave was completely excavated to a depth of 70 cm prior to donor interment and infilling, the observed differences in richness along transects and with respect to both the top of the grave and the grave controls suggests that physical processes and mixing may be partly responsible for the observed community change. Indeed, studies of agricultural soils show that cultivation can reduce nematode abundance and diversity [54].

With few exceptions [84–86], most field studies of nematodes focus on a single depth range, usually within the top 30 cm of the soil profile [38,47,49,52,53,56,87]. Our study compared multiple depths, and revealed that abundance, richness, and diversity decreased with depth both within and outside the grave. Within the grave, there was an overall reduction in nematode abundance and richness with depth. Previous studies have reported that approximately 70% of nematodes exist in the top 20 cm of soil and their distributions are associated with available food sources [85,86]. Our findings of elevated abundances at 30‒35 cm, in conjunction with high variation in samples at those depths, is likely due to elevated bacterial and fungal abundances in these areas. The lowest abundance, richness, and diversity were observed at the base of the grave (70‒75 cm). Nematodes were not detected below the grave (85‒90 cm), which is consistent with observations of nematode abundances found in deep agricultural and forest soils [85].

The faunal profile of the nematode communities provided some insight to their response to physical disturbance and decomposition products (Fig 6). Transect surface soils largely fall within quadrant A, indicative of enrichment by opportunistic taxa. Transect soils from 30‒35 cm are shifted toward quadrant B, indicative of increasing community maturity with undisturbed depth. Surface grave soils also fall within quadrant A; however, they are clustered below 40 on the SI, indicating that severe physical disruption caused by excavation and infilling influenced nematode populations. Similarly, 30‒35 cm soils within the grave are shifted lower on the SI compared to transect soils of the same depth. The observation that soils in close proximity to decomposition products display a difference in community structure is consistent with observations by Szelecz et al. [38]. Meng et al. [85] reported that most enrichment opportunists (B1 taxa) were found to inhabit the upper 20 cm of the soil profile, but that the location of nematodes in a vertical profile was dependent upon food source location. In our study, the location of decomposition products and associated microbial blooms clearly influenced nematode communities below 20 cm.

Interestingly, within 30‒35 cm grave soils, one sample was strongly enriched with a unique (unidentified) species of *Plectus* (B2) that was found only within the grave. Modest increases in bacterial abundances can stimulate some B2 taxa without exceeding threshold stimulation for enrichment opportunist response (B1 taxa) [88]. This may account for the surprisingly low abundances of bacterial feeding enrichment opportunists (notably Rhabditidae). Samples from 70‒75 cm depths within the perched water table had extremely low SI values and highly variable EI values, indicating a wide range in enrichment and disturbance status of these soils. These samples were low in abundance (n = 25 ± 26) and richness (n = 4 ± 4), resulting in high variation in EI. Members of c-p classes 3‒5, which include K-selected taxa, were not observed, thus accounting for the low SI. It is possible that decomposition products and anaerobic conditions may have created sufficient chemical disturbance to inhibit nematodes sensitive to environmental change, and thus skew these communities in favor of taxa in c-p classes 1 and 2.

*Plectus* has been proposed as a potential indicator taxon for chemical disturbances. It has been shown to negatively correlate to chemical fertilization; however, it positively correlates with organic amendments [54,89]. We found *Plectus* to be enriched within the grave (both 30‒35 cm and ribcage samples) with a concomitant depletion of *Filenchus* abundances. This was in contrast to all other soils where *Filenchus* was more abundant than *Plectus*. *Plectus* and *Filenchus* are both classified as general opportunists (c-p 2), but occupy different trophic groups and so do not compete for resources. To our knowledge, this is the first observation of a potential inverse relationship between *Filenchus* and *Plectus*, suggesting either a potential interaction between them or contrasting sensitivities. Further study is warranted as these taxa may be useful as indicator taxa for determining decomposition progress.

The mixed soil and adipocere sample collected within the ribcage of one donor contrasted sharply with nearby soil biogeochemistry and microbial ecology. In particular, total DNA, bacterial and fungal copy numbers, and microbial respiration rates were higher than any other soil collected within or outside of the grave. Microbial LAP and PDE enzymatic activity was also elevated, contrasting with the soils collected above and below the donors. Interestingly, despite the sample coming from inside a body cavity, there were no human-associated *Bacteroides* gene copies detected. Nematodes from the genus *Plectus* were also enriched in this sample compared with transect soils. The elevated respiration, abundant bacterial-feeding *Plectus*, and elevated enzymatic activity specific for protein and nucleic acid degradation suggests that C and N breakdown from carcass-derived organics was still actively occurring four years post-burial.

## Conclusions

Subsurface human decomposition physically and chemically altered the surrounding soils in this system. After four years of burial in clay-rich sediments at the ARF, soils still preserved several physiochemical and biological indicators of interment and decay. At the base of the grave, elevated respiration rates and microbial gene abundances demonstrated that microbial communities were still present and active–likely because decomposition was active and on-going. Human-associated *Bacteroides* persisted at the base of the grave and demonstrated the potential for these microbes to serve as markers of human decomposition in the subsurface environment, at least in a system with protracted decay. Extracellular enzyme activity specific for chitin degradation (NAG) was elevated. The microbial communities persisting at the base of the grave are able to utilize more recalcitrant C and N pools, likely driven by low oxygen availability. Soils were also elevated in DON, DOC, and ammonium, demonstrating that indicators of decomposition persisted. Nematode communities were affected by the presence of decomposing remains, supporting the idea that they may be potential indicators of carcass-derived nutrient enrichment in soil.

Given the constraints associated with human taphonomy research, and the destructive nature of the sampling (i.e. excavation), this study was not replicated (i.e. included only one test grave and one control grave) and does not include a time series examination of these changes. Future work on the repeatability of these results and the temporal dynamics will be needed to extrapolate beyond this system and build a consensus of the impacts of buried remains on soils.

## Supporting information

S1 Table Biometric data of deceased human subject donors used in the study. (XLSX)

S2 Table Measured soil physicochemical and biogeochemical parameters for all samples taken during the study. Summary statistics of the data are presented in Table 1. Off-grave control samples from an undisturbed location are indicated with *; Samples from the control grave (excavated but no donors interred) are indicated with ⱡ. DON = dissolved organic nitrogen; DOC = dissolved organic carbon.
(XLSX)

S3 Table Measured soil enzyme activities for all samples taken during the study. Sample descriptions (location and collection date) are provided in S2 Table. Mean and standard deviations of these data are shown in Table 1 and Fig 3. Off-grave control samples from an undisturbed location are indicated with *; Samples from the control grave (excavated but no donors interred) are indicated with ⱡ. PDE = phosphodiesterase activity; NAG = N-acetyl-β-glucoseaminidase activity; CB = β-D-cellulobiosidase activity; LAP = leucine aminopeptidase activity; COL = collagenase activity.
(XLSX)

S4 Table Counts of nematode genera from soils collected along transects at the surface (0 cm depth). Columns represent samples along transects at 0.5, 1.0, 1.5, and 2.0 m from grave. *Off-grave control samples.
(XLSX)

S5 Table Counts of nematode genera from soils collected along transects at the surface (30 cm depth). Columns represent samples along transects at 0.5, 1.0, 1.5, and 2.0 m from grave. *Off-grave control samples.
(XLSX)

S6 Table Counts of nematode genera from soils collected within the grave. Columns represent samples from different depths within the grave. *Control grave samples.
(XLSX)

S1 Fig Aerial image of study site. The multi-individual grave is indicated by “3”, and the control grave by “C”. The other two boxes (6 and 1) represent other graves not used in this study. Arrow indicates North. Figure adapted from [90].
(PDF)

S2 Fig Changes in soil physicochemical parameters within the grave. Points are mean and standard deviation of n = 3 samples.
(PDF)

S3 Fig Piper diagram for grave water and nearby surface water geochemistry. Water samples from five East Tennessee rivers and the grave water are geochemically similar, and are predominantly Ca-Na-K-HCO_3_^-^ type waters. Surface water geochemistry was obtained through the USGS [91], selecting samples from nearby rivers (less than 50 miles away), with complete water geochemistry provided (including pH, temperature, total dissolved solids, bicarbonate), and ionic charge balance of +/- 17%. The resulting dataset included 40 surface samples plus the grave water.
(EPS)

S4 Fig Gene copy abundances of Total 16S rRNA (Bacteria), *Bacteroides* 16S rRNA (*Bacteroides*) and ITS (Fungi) within the grave. *Bacteroides* were below detection in all but the 70‒75 cm samples.
(PDF)
