# Supplementary material for: Spatial impacts of a multi-individual grave on microbial and microfaunal communities and soil biogeochemistry
Source: PLoS One. 2018 Dec 12;13(12):e0208845. doi: 10.1371/journal.pone.0208845 (PMC6291161; doi:10.1371/journal.pone.0208845)
Supplement: S1 Fig — The multi-individual grave is indicated by “3”, and the control grave by “C”. The other two boxes (6 and 1) represent other graves not used in this study. Arrow indicates North. Figure adapted from [90]. (PDF) [file pone.0208845.s007.pdf]

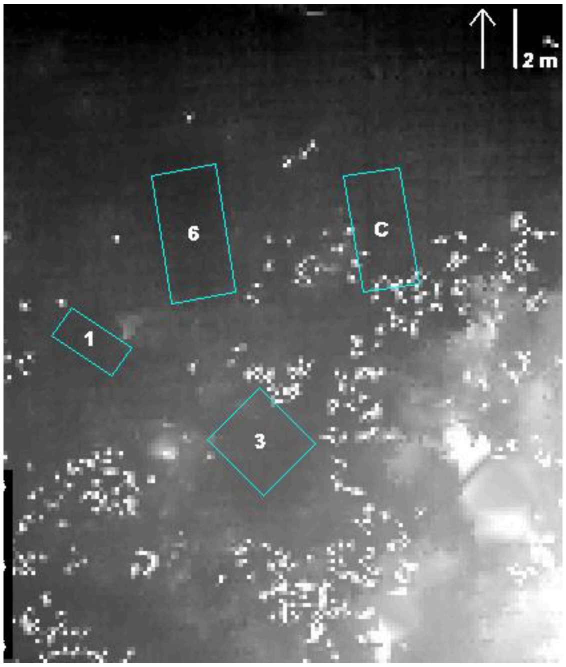

S1 Fig. Aerial image of study site. The multi-individual grave is indicated by “3”, and the control grave by “C”. The other two boxes (6 and 1) represent other graves not used in this study. Arrow indicates North. Figure adapted from [90].
