# Supplementary material for: Spatial impacts of a multi-individual grave on microbial and microfaunal communities and soil biogeochemistry
Source: PLoS One. 2018 Dec 12;13(12):e0208845. doi: 10.1371/journal.pone.0208845 (PMC6291161; doi:10.1371/journal.pone.0208845)
Supplement: S2 Fig — Points are mean and standard deviation of n = 3 samples. (PDF) [file pone.0208845.s008.pdf]

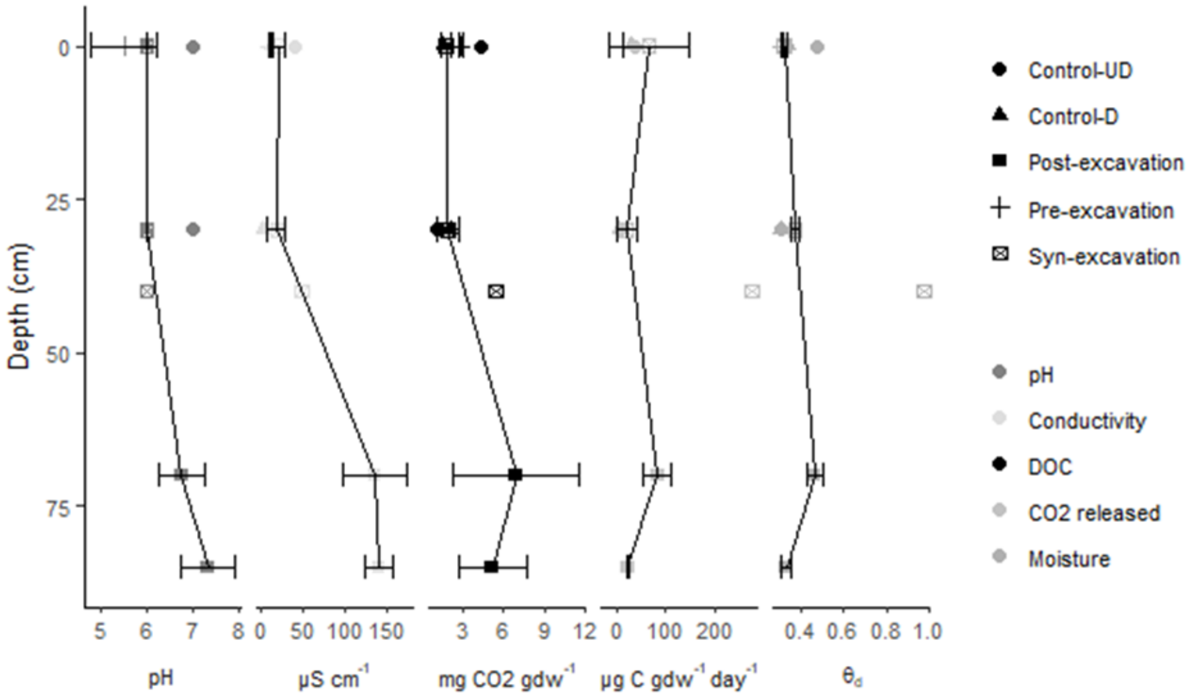

S2 Fig. Changes in soil physicochemical parameters within the grave. Points are mean and standard deviation of n=3 samples.
