# Supplementary material for: Spatial impacts of a multi-individual grave on microbial and microfaunal communities and soil biogeochemistry
Source: PLoS One. 2018 Dec 12;13(12):e0208845. doi: 10.1371/journal.pone.0208845 (PMC6291161; doi:10.1371/journal.pone.0208845)
Supplement: S4 Fig — Bacteroides were below detection in all but the 70‒75 cm samples. (PDF) [file pone.0208845.s010.pdf]

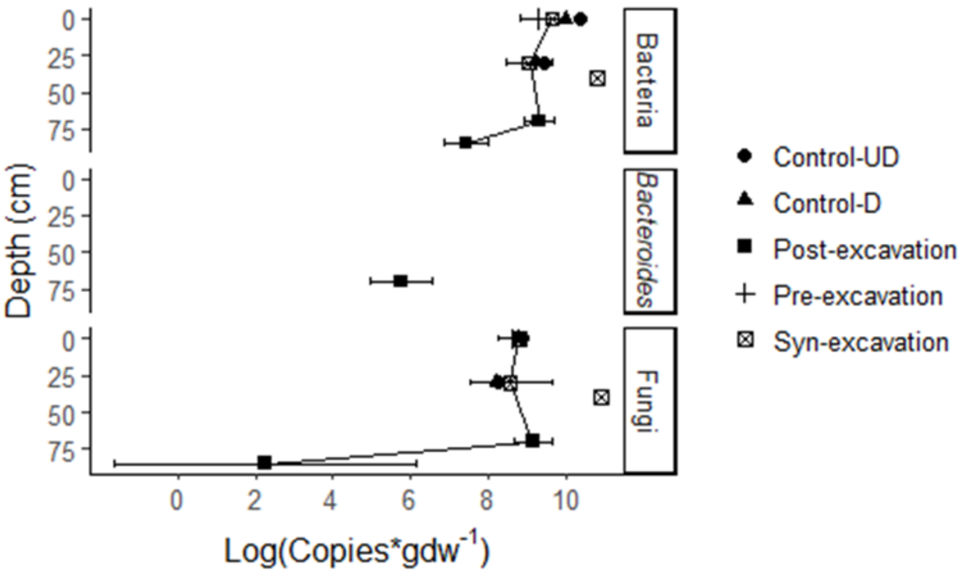

S4 Fig. Gene copy abundances of Total 16S rRNA (Bacteria), *Bacteroides* 16S rRNA (*Bacteroides*), and ITS (Fungi) within the grave. *Bacteroides* were below detection in all but the 70-75 cm samples.
